# Long-term results of surgical management high-energy scapula fractures at a major trauma center

**DOI:** 10.1016/j.jseint.2026.101682

**Published:** 2026-03-05

**Authors:** Shoib Mahmood, Georgios Mamarelis, Mochamed Hachem, Adel Tavakkolizadeh, Karthik Karuppaiah, Toby Colegate-Stone

**Affiliations:** Department of Trauma and Orthopaedics, King's College Hospital, London, UK

**Keywords:** Scapula fracture, Shoulder trauma, Intra-articular fracture, Scapular fixation, Post-traumatic arthrosis, Patient-reported outcomes, Orthopedic trauma, Major trauma center

## Abstract

**Background:**

Scapular fractures are rare injuries typically resulting from high-energy trauma and often occur in polytrauma patients. Historically managed nonoperatively, recent trends in surgical intervention have evolved, particularly in major trauma centers (MTCs) where expertise and case volume have increased. However, there remains limited literature on the surgical management and long-term outcomes of these injuries.

**Methods:**

This retrospective cohort study reviewed all patients who underwent surgical fixation for scapular fractures over a 10-year period at a single UK MTC. Patient demographics, injury mechanism, fracture classification (using the Swedish Fracture Registry topographical system), surgical approach, and outcomes were analyzed. Outcomes included objective measures (union, complications, return to work/sport) and patient-reported outcome measures (Oxford Shoulder Score, disability of the arm, shoulder, and hand, American Shoulder and Elbow Surgeons score, EuroQol 5-Dimension questionnaire, and EuroQol Visual Analogue Scale). Statistical analysis included chi-squared and Mann-Whitney-Wilcoxon tests.

**Results:**

Forty-five patients (36 males; mean age 46 years) were included. The majority (64%) sustained injuries from road traffic accidents, with 49% classified as having severe or profound injuries (injure severity score >15). Displaced intra-articular fractures represented the most common fracture subtype (53%). At a mean follow-up of 78 months, 56% of patients completed patient-reported outcome measures. Mean scores were Oxford Shoulder Score: 40.1, disability of the arm, shoulder, and hand: 15.3, American Shoulder and Elbow Surgeons score: 82.7, EuroQol 5-Dimension questionnaire: 0.54, and EuroQol Visual Analogue Scale: 82.1. Intra-articular fractures were associated with the highest complication and reoperation rates, primarily due to post-traumatic glenohumeral capsulitis and early arthrosis. The overall complication rate was 35.7%, reducing to 15.6% when excluding capsulitis. No 30-day mortality was observed. Return to work and sport occurred at a mean of 147 and 185 days, respectively.

**Conclusion:**

Surgical management of high-energy scapular fractures in a high-volume MTC setting can achieve good long-term clinical outcomes. Intra-articular fracture patterns carry a higher risk of complications and may require further intervention. This study provides the longest reported follow-up of scapula fracture surgery to date and highlights the importance of specialist surgical experience, structured classification, and long-term patient support.

Scapular fractures are a rare but potentially complex injury. Scapula fractures constitute about 0.5-1% of all fractures and 3-5% of upper limb injuries.^3^^,^^8^ They are often sustained secondary to high-energy trauma as such present in patients with polytrauma. As such patients may need to be funneled to major trauma centers (MTCs). The current literature regarding the surgical management and outcomes regarding this group is limited.^1^^,^^2^^,^^5^^,^^8^^,^^12^^,^^13^ Traditionally, most extra-articular scapular fractures have been treated conservatively with the expectation that shoulder function will be minimally affected.^14^ Such strategy is less favorable in notably displaced injuries or in combination with significant intra-articular fractures, notable glenoid rotation, or shoulder suspensory complex type injuries. In the UK prior to the introduction of the MTC network, surgical experience and training was limited regarding such cases. Historically, each center would manage their own cohort, in turn, limiting surgeon experience and operative case load. Patients with complex scapula injuries remain a challenging cohort to successfully treat as they often not only have these shoulder girdle injuries but also other associated high acuity polytrauma. Such patients are now often referred to MTCs and, in turn, surgical experience has evolved as volume has increased.

The aim of this retrospective cohort study was to evaluate a decade of surgically managed scapula fractures at a single high-volume UK MTC. Specifically, we sought to describe the fracture characteristics that led to operative intervention, the surgical strategies employed, and associated clinical and complication outcomes. A secondary aim was to correlate outcomes with scapular fracture topography. Rather than re-evaluating the general indications for operative fixation—which are well established for selected patterns—this study focuses on characterizing the spectrum of fracture subtypes treated surgically in contemporary practice. We hypothesized that operative management is predominantly reserved for specific fracture patterns, particularly articular and complex configurations, where nonoperative management is less likely to provide predictable outcomes.

## Material and methods

A review was undertaken regarding any surgical fixation of a scapula fracture undertaken at the single MTC over the preceding 10 years. A database was constructed to outline patient demographics, mechanism of injury, scapula fracture topography, associated injuries, injury severity score (ISS), outcomes, and complications. The study followed the requisite institutional requirements and advice.

**Figure 1:**
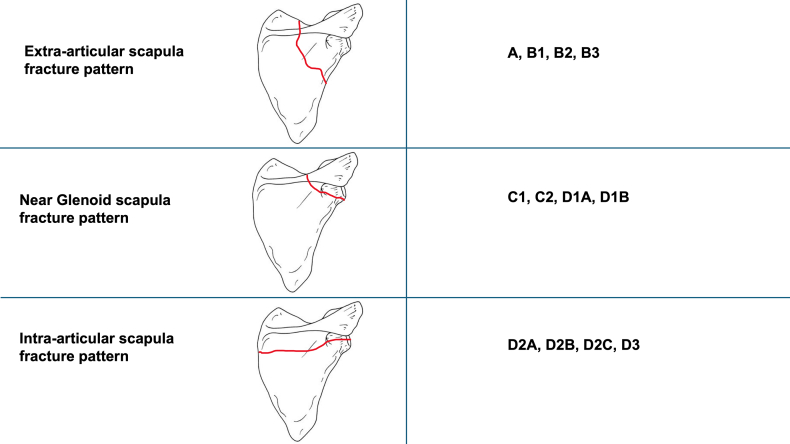
Classification in the Swedish Fracture Register (SFR) with A-C (extra-articular) classified according to the Euler-Ruedi classification and D (intra-articular) classified according to the Ideberg classification.

All patients had shoulder girdle computed tomography scans prior to surgery to aid decision-making. This also enabled scapula fracture topography to be accurately assessed. Extra-articular fractures are classified according to the Euler-Ruedi classification, intra-articular fractures according to the Ideberg classification. Using these as the basis for categorization, the Swedish Fracture Registry describes 12 scapula fracture classes (Fig. 1). These 12 classes are, in turn, grouped into 3 more simplified scapula fracture subtypes—extra-articular, glenoid near, and intra-articular. These are defined as follows: extra-articular body (A: body), process (B1-B3: scapular spine, coracoid process, acromion); glenoid near fracture: neck (C1-C2: anatomic and surgical glenoid neck), glenoid rim (D1a-D1b: anterior and posterior glenoid rim, ie, Ideberg type I); intra-articular glenoid (D2a-D3: intra-articular glenoid fracture with scapular exit inferior, horizontal, superior, or comminuted, ie, Ideberg type II-V).

The population was assessed using this simplified classification system and subdivided the patients in these 3 different topographical groups (intra-articular; near glenoid; extra-articular).

Patients were identified retrospectively from institutional trauma and operative theater databases at a UK MTC. All adult patients undergoing operative fixation of a scapula fracture during the study period were eligible for inclusion. Patients managed nonoperatively were not included, as the aim of this study was to characterize fracture patterns, operative indications, and outcomes in surgically treated patients rather than to compare treatment strategies.

### Outcomes

Clinical outcomes for all patients were assessed both objectively and subjectively. The objective outcomes assessed were achievement of bony union and need for further intervention. Subjective outcome assessment was performed using the Oxford Shoulder Score (OSS) questionnaire, disability of the arm, shoulder, and hand (DASH), American Shoulder and Elbow Surgeons (ASES) score and the EuroQol 5-Dimension questionnaire (EQ-5D), and EuroQol Visual Analogue Scale (EQ-VAS).^4^^,^^6^^,^^7^^,^^9^

All assessment tools aim to provide an assessment of the overall outcome through the questions relating to pain, function, and psychological well-being. An OSS of less than 20 indicates poor shoulder function, whereas a score of 48 is normal. The DASH score is out of 100 with zero representing no disability and 100 the maximum disability. A DASH sore of over 50 represents severe disability. The ASES is out of 100 with zero representing the worst function and pain and 100 no pain and excellent function. An ASES score of less than 50 represents poor shoulder function. As such, these tools enabled an estimation of an individual's level of pain, function, and psychological well-being at the end of their care pathway.

Other objective clinical outcomes were assessed including complications, need for secondary procedures, time to return to work, time to return to sport, and mortality.

### Statistics

Statistical analysis was performed. Chi-squared testing was performed to assess the relationship between nominal categorical variables. Mann-Whitney-Wilcoxon testing was performed to assess the relationship between continuous nonparametric data (OSS, ASES, DASH EQ-5D, and EQ-VAS scores). A *P* value of .05 or less was considered significant.

## Results

### Demographics and fracture history

Forty-five patients were included (36 males, 9 females; mean age 46 years, age range 14-70 years). The commonest mechanism of injury was road traffic accident (64%, n = 29) (Table I). The majority of patients (58%; n = 26/45) were previously fit (American Society of Anesthesiologists 1) without significant medical histories. Associated orthopedic injuries and additional system injuries were noted in over half the cohort. ISS analysis was conducted. The ISS assessment split the cohort into the following groups: mild (<9), moderate (9-15), severe (16-24), and profound (>24) (Table I). Patients with an ISS >15 are associated with an approximate risk of mortality of 10%. Almost half (49%) of the patients were classified as severe or profoundly injured with an ISS >15. Significant chest injuries were noted in 51% of patients.

**Table I:** Summary of observed cohort characteristics.

Variable	Subgroup	Number of patients (n)	Percentage (%)
Mechanism of injury	Bicycle	10	22%
	RTA	29	64%
	Fall from ladder	1	2%
	Fall down flight of stairs	2	4%
	Tree falling onto patient	1	2%
	Assault	1	2%
	Fall from balcony	1	2%
Other orthopedic trauma injuries	Orthopedic – pelvis (n = 3); spinal (n = 9); long bone (n = 7); others (n = 11)	25/45	56%
Other system injuries	Head (n = 9); chest (n = 23); abdominal organ (n = 3)	27/45	60%
Index injury-related neurovascular injury		7	15%
ISS	Mean Range	16.4 4-50	
	Minor	0-16	n = 23
	Serious	17-24	n = 10
	Severe	25 and over	n = 12

*ISS*, injury severity score; *RTA*, road traffic accident.

The majority, 24/45 (53%), of the patients sustained displaced intra-articular fracture patterns (Table II). There were high rates seen of other shoulder girdle injuries including acromioclavicular joint (n = 7), clavicle fractures (n = 10), and proximal humerus fractures (n = 4). The Superior Shoulder Suspensory Complex (SSSC) is essential in maintaining the stability of the shoulder girdle, ensuring good biomechanics and a stable connection between the upper extremity and the axial skeleton. From the cohort, 27/45 (60%) had a SSSC type injury. If the scapula classifications (Euler-Ruedi and Ideberg classifications) were strictly adhered to, over half, 25/45 (56%), would not fit exactly into the definitions of these sub types. As such, the adapted Swedish Fracture terminology regarding extra-articular, near glenoid, and intra-articular best suited fracture topography description and communication regarding this study cohort. Presurgery neurological injury was noted in 7/45 (15%) of cases.

**Table II:** Summary of scapula fracture topography.

Extra-articular fracture pattern	n = 13	N	29%
	A	0	
	B1	2	
	B2	9	
	B3		

Near glenoid fracture pattern	n = 8		18%

	C1	0	
	C2	7	
	D1A		
	D1B		

Intra-articular fracture pattern	n = 24		53%

	D2	7	
	D3	13	
	D1A	2	
Scapula & humerus fracture	E	4	
SSSC		27	60%
ACJ injury Clavicle fracture		7 10	15% 22%

*SSSC*, Superior Shoulder Suspensory Complex; *ACJ*, acromioclavicular joint.

### Delivered care

**Figure 2:**
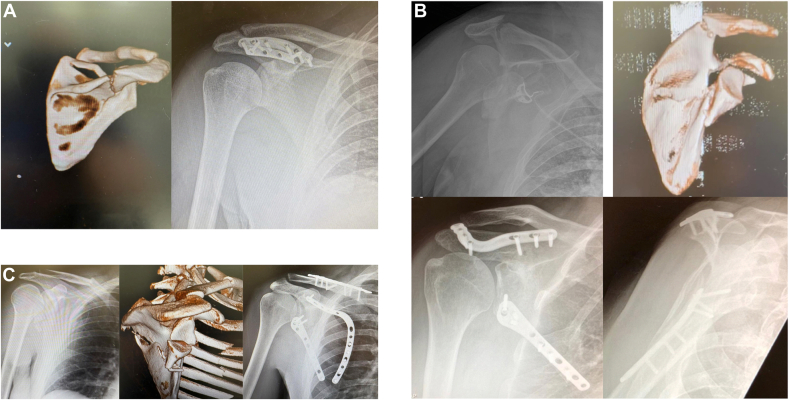
(**A-C**) Preoperative and postoperative scapula fracture fixation imaging for 3 patterns: extra-articular (**A**); near glenoid (**B**); intra-articular (**C**).

Surgery was performed using a standardized approach. For anterior-based intra-articular and glenoid rim–based fracture patterns, an anterior deltopectoral type approach was used for access. In addition to and prior to the open approach, an arthroscopic assessment was additionally undertaken. Overall, a deltopectoral anterior approach was used in 13/45 (29%) of cases. For posteriorly based intra-articular and near glenoid fractures, a posterior approach was utilized. This was either through a modified Judet approach or a formal Judet depending on the requirements of any additional scapula body or process related injuries. If possible, a modified judet approach was preferred. Posteriorly based surgical approach was used in 24/45 (53%) of cases. Regarding solely extra-articular fractures, the approach was dependent on the location of the fracture and any other associate SSSC type injury. Isolated scapula spine fractures were approached directly as were isolated acromial fractures. Associated clavicle and proximal humeral fractures were accessed via separate incisions as required. Fracture fixation usually utilized precontoured shoulder girdle plates (Fig. 2 *A*–*C*).

### Clinical outcomes

**Figure 3:**
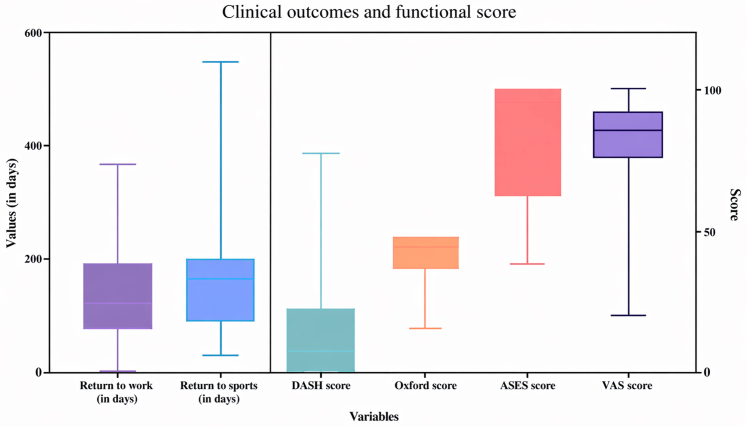
Clinical outcomes and functional scores.

Outcomes were collected at a mean 78 months (range 13-144 months). Patient-reported outcome measure (PROM) scores were collected from 56% of patients (n = 25/45). The mean OSS, DASH, ASES EQ-5D, and EQ-VAS scores for the whole cohort at final follow-up were 40.1,15.3, 82.7, 0.54, and 82.1, respectively (Table III & Fig. 3). This was then assessed in subgroups. When the study population's OSS at the end of care were compared against the average accepted score of a poorly functioning shoulder, the patients in this study had a significantly better (*P* < .05) outcome as against such a category. Subgroup analysis of the cohort by fracture topography (extra-articular fracture/near glenoid fracture/intra-articular fracture) demonstrated no significant difference in outcome as assessed by OSS, DAS, ASES, EQ-5D, or EQ-VAS.

**Table III:** Summary of patent-reported outcomes.

Fracture type	OSS	DASH	ASES	EQ-VAS	ISS	Further procedures
Extra-articular fracture pattern N = 13	45.2	27.8	96	65	17	2
Intra-articular fracture pattern N = 12	40.5	16.3	80.6	84.7	14	9
Near glenoid fracture pattern N = 8	37.8	8.6	82	84	26	3

*OSS*, Oxford Shoulder Score; *DASH*, disability of the arm, shoulder, and hand; *ASES*, American Shoulder and Elbow Surgeons score; *ISS*, injury severity score; *EQ-VAS*, EuroQol Visual Analogue Scale.

The most common local postoperative complication was post-traumatic glenohumeral capsulitis (23.8% n = 10/42) (Table IV). This was managed through a sequential approach including physio, ultrasound-guided glenohumeral joint injection, and if this was not sufficient glenohumeral capsular release and arthrolysis. Removal of metalwork was performed in 8 patients; revision fracture fixation was required in 1 case. Overall, further surgeries were required in 14 patients (31%). New postoperative neurological injury was noted in only 1 patient. This was a neurapraxia to the musculocutaneous nerve, which resolved in time. Currently, 3 patients have developed early symptomatic post-traumatic arthrosis, but none have required arthroplasty surgery to date as symptoms manageable with conservative measures, and all 3 are young individuals. There were no cases of deep infection. There were no cases of acute 30-day mortality; however, at follow-up, 2 patients were deceased. The causes were unrelated with one being secondary to esophageal cancer and the other liver cirrhosis. If all cases of post-traumatic capsulitis were included, then the overall complication rate was 35.7% (n = 15/42); however, if these were excluded, the rate fell to 15.6%. The average time to return to work for those who were able was 147 days postinjury (range 4-365 days), while the average time to return to sport for those who were able was 185 days (range 30-548 days).

**Table IV:** Summary of recorded complications.

Complication	N	% (of 42)
Overall complication rate	15/42	35.7%
Revision fixation	1	2.4%
Removal of metalwork	8	19.0%
Postoperative nerve injury	1	2.4%
Deep postoperative infection	0	0%
Superficial stitch abscess	1	2.4%
Post-traumatic glenohumeral capsulitis	10	23.8%
Delayed ACJ reconstruction	1	2.4%
Broken metalwork	1	2.4%
Symptomatic ACJ arthrosis	1	2.4%
Heterotopic ossification	1	2.4%
Nonunion	1	2.4%
Postoperative VTE	1	2.4%
Long-term mortality	2	4.8%

*ACJ*, acromioclavicular joint; *VTE*, venous thromboembolism.

When complications and further operations were viewed from the perspective of the different main scapula fracture subtypes, those with intra-articular fractures had the highest rates of complications (Table III). The main cause for further operation in the intra-articular subgroup was post-traumatic glenohumeral capsulitis and arthrosis, with the procedures being undertaken relating to this including the following: guided glenohumeral joint injection, arthroscopic capsular release and arthrolysis, and removal of metalwork. In the extra-articular subgroup, the further procedures included removal of acromion metalwork. In the near glenoid subgroup, the further procedures related to peripheral nerve injury surgery (with injuries sustained from the index accident and not the surgery) and post-traumatic glenohumeral capsulitis and removal of metalwork.

## Discussion

This study demonstrated that patients with high-energy displaced scapula fractures can achieve good clinical outcomes following appropriate surgical reconstruction and rehabilitation. However, these patients can have a prolonged course of recovery with a notable requirement for further intervention. This is usually associated with the management of post-traumatic glenohumeral capsulitis and arthrosis. When scapula fractures are assessed from a topographical perspective, those with displaced intra-articular fractures have the highest rates of developing postoperative complications and need for further intervention. Good outcomes were noted following the fixation of displaced extra-articular fractures. These patients may need subsequent removal of metalwork postunion secondary to the relatively subcutaneous positioning.

Higher rates of other orthopedic injuries as well as other system injuries were recorded in this population as compared to other studies.^11^^,^^13^ All the patients in this study had high-energy injuries. Such trauma often does not just injure the shoulder girdle but also impacts others systems as demonstrated by over half of the patients sustaining a significant chest injury as well as the high ISS. Almost half of the studied cohort being classified by ISS as having a serious or severe life threatening set of injuries. This highlights the need for the focus of care to be at a MTC. This helps to explain why such a concentration of cases was gathered in 1 unit, which helped to develop the surgical experience and techniques.

Not all scapula fractures require fixing. There exists good evidence for the nonoperative management of scapula fractures. For example, those with low energy and minimally displaced fractures can achieve excellent outcomes with conservative treatment modalities. This is particularly the case in those with extra-articular scapula body injuries.^11^^,^^14^ The largest assessment in the literature of scapula fractures was undertaken in Sweden utilizing the Swedish Facture Registry. This included approximately 4,000 scapula fracture patients and reported a 81% nonoperative management rate.^11^ However, in that study, only 22% of injuries were classified as high-energy trauma, while all the cases in the present study were high-energy displaced fracture patterns, hence, necessitating a surgical approach. The observed population demographics were of male predominance, as noted in other studies.^11^^,^^13^ Perhaps, an indication of a skewed predilection for higher risk taking behavior. Associated injuries were seen to be higher in the described population with higher ISS scores as compared to other studies.

There exists good outcome evidence for the surgical management of displaced scapula fractures.^10^^,^^11^^,^^13^^,^^14^ This includes those with intra-articular, near glenoid fracture patterns, and extra-articular topography. Current indications for surgery include significantly displaced intra-articular fractures >5 mm, significant scapular blade angular deformation of more than 45°, medial or lateral glenoid displacement of over 2 cm, double disruption of the SSSC, Glenopolar angle (GPA) less than 22° (defined as the angle between the line connecting the uppermost with the lowermost point of the glenoid cavity and the line connecting the uppermost point of the glenoid cavity with the lowermost point of the body of the scapula), and open fractures.^1^^,^^2^^,^^11^ The injuries operated on in this cohort met such criteria. This study demonstrates a need for a surgical service that is experienced and able to deal with complex scapula injuries. Displaced intra-articular injuries formed the majority of the cases operated on; however, there was a high rate of those with SSSC injuries or displaced extra-articular patterns.

Having a biomechanically stable scapula is important in all patients but of particular relevance in the younger working and sport active population. Considering the long-term complications of scapula and glenoid-related fractures is equally important, which can result in need for shoulder arthroplasty surgery at a later date. Glenoid implant and reconstruction options are optimised in patients with preserved glenoid orientation and adequate bone stock, whereas outcomes are less favourable in those with chronically neglected injuries and associated bone loss and deformity. Arthroplasty in these neglected scenarios becomes far more challenging with potentially poorer outcomes as compared to those with better aligned anatomy. Indeed, the timing for arthroplasty can potentially be accelerated based on severity of injury and progression of arthrosis. Of note, while there are examples in this studied cohort with early post-traumatic glenohumeral arthrosis, they have yet to progress to joint arthroplasty, which is of relevance given the younger age of the studied patients. As such, reconstruction of scapula fractures can optimize the outcome and delay the need for arthroplasty in majority of patients. This is of particular relevance given the known higher revision rates in the younger patients with arthroplasty. Arthroplasty outcomes are often negatively impacted by the additional soft tissue as well as bony injuries, potentially needing more complex revisions earlier on with the associated concerns regarding higher morbidity, poorer outcomes, and increased costs.

The surgical strategy employed in this cohort aimed to avoid the full posterior Judet approach unless completely necessary, as this approach requires an extensive soft tissue dissection. Modified posterior Judet approach can often provide adequate exposure. There is a relative paucity of specialist scapula plates. In the absence of specialist kit, it is possible to manage some scapula fractures with nonanatomical plates. Screw length in this scenario needs some consideration as the scapula can be thin necessitating smaller screw lengths. As such, it is important to plan carefully prior to the surgery and to have a clear strategy and to have a variety of kit options and implants available. Operating as a paired consultant team can develop experience and enable the best intraoperative decision-making. Such an approach is recommended by UK national bodies and is good to build case volume when embarking on these complex interventions that carries known and inherent risks.^15^ Arthroscopic assessment of selected glenoid avulsion-type injuries can be a helpful adjunct prior to an open anterior approach. However, its role is limited in the presence of significant comminution. In this study, arthroscopy was utilized selectively for isolated inferior-based glenoid sleeve injuries. Given the technical demands of this procedure, its use requires an experienced surgeon and prompt intraoperative decision-making, particularly when an extensive open approach is also required to facilitate definitive fixation.

There is accepted variation in the prognosis of different scapula injuries. The expectation would be a worse outcome in those cases with intra-articular injuries with comminution, bone loss, soft tissue loss, and delays to management. Interestingly, this series demonstrates the final outcomes to be more positive than expected; however, the outcomes can fail to reach the normal preinjury scores. It is important to emphasize to the patient early on in their care that the end-to-end pathway can be lengthy and often requires multiple procedures in order to achieve the optimal outcome. It is difficult to directly compare this series as there are no others available in the literature.

The objective and subjective outcomes demonstrated in this study indicate good results in all 3 main subgroups: extra-articular, intra-articular, and near glenoid. However, it is suggested that the patients should be appropriately advised of the range of outcomes with conservative and surgical approaches and the notable risks of requiring further intervention at a later date with particular risk of developing post-traumatic capsulitis and need for removal of metalwork. If patients requiring treatment for capsulitis and removal of metalwork were excluded the observed complication rate dropped to 13%. This study is the longest reported follow-up on scapula fractures. However, there is a relative paucity of studies. The observed mortality rate is lower than expected or reported elsewhere. This is despite the significant other injuries and the high ISS >16.^16^ This may reflect the experience and impact a specialist high volume MTC has in these high acuity cases.

### Limitations

This study has several limitations. PROMs should be interpreted with caution, as no nonoperative comparator group was included; meaningful comparative analysis (eg, operative versus nonoperative DASH scores) is therefore not possible, and PROMs are presented descriptively only. PROM data were available for approximately half of the cohort, introducing potential response bias.

Selection bias is inherent, as only surgically treated scapular fractures were included and the study was not designed to evaluate treatment indications or compare management strategies. D1a/b fractures were included only when treated operatively and often represent a distinct clinical entity managed according to the dominant pathology rather than the scapular fracture itself. Complete long-term follow-up could not be ensured for all patients, particularly those referred from outside the local catchment area, and late complications may have been missed. The cohort was relatively small and heterogeneous, procedures were performed by multiple consultants, and the findings apply only to selected operatively treated patients, limiting generalizability.

## Conclusion

Displaced scapula fractures secondary to high-energy trauma often present with notable ISS and other significant orthopedic and multisystem injuries. As a result, the timelines for definitive fixation can be delayed. Despite this, good end outcomes can be achieved but can take a protracted course. Subgroup analysis indicates that those with intra-articular fractures are more likely to develop postoperative glenohumeral capsulitis or arthrosis. In turn, this subtype has a higher rate of complication and need for additional procedures at a later stage. This is most commonly in the form of removal of metal work and arthrolysis. A subspecialist unit and an experienced team-based surgical approach are advocated whenever possible. This study offers the longest follow-up outcomes in the literature to date.

## Disclaimers:

Funding: No funding was disclosed by the authors.

Conflicts of interest: The authors, their immediate family, and any research foundation with which they are affiliated have not received any financial payments or other benefits from any commercial entity related to the subject of this article.
